# Nucleotide alterations in the HLA-C class I gene can cause aberrant splicing and marked changes in RNA levels in a polymorphic context-dependent manner

**DOI:** 10.3389/fimmu.2023.1332636

**Published:** 2024-01-24

**Authors:** Akiko Mizutani, Shingo Suzuki, Atsuko Shigenari, Tadayuki Sato, Masafumi Tanaka, Jerzy K. Kulski, Takashi Shiina

**Affiliations:** ^1^ Department of Molecular Life Science, Tokai University School of Medicine, Isehara, Kanagawa, Japan; ^2^ Faculty of Health and Medical Science, Teikyo Heisei University, Tokyo, Japan; ^3^ School of Biomedical Sciences, The University of Western Australia, Nedlands, WA, Australia

**Keywords:** human leukocyte antigen, HLA Class I genes, polymorphisms, alternative splicing, RNA levels, ectopic expression assay

## Abstract

Polymorphisms of HLA genes, which play a crucial role in presenting peptides with diverse sequences in their peptide-binding pockets, are also thought to affect HLA gene expression, as many studies have reported associations between HLA gene polymorphisms and their expression levels. In this study, we devised an ectopic expression assay for the HLA class I genes in the context of the entire gene, and used the assay to show that the HLA-C*03:03:01 and C*04:01:01 polymorphic differences observed in association studies indeed cause different levels of RNA expression. Subsequently, we investigated the C*03:23N null allele, which was previously noted for its reduced expression, attributed to an alternate exon 3 3’ splice site generated by G/A polymorphism at position 781 within the exon 3. We conducted a thorough analysis of the splicing patterns of C*03:23N, and revealed multiple aberrant splicing, including the exon 3 alternative splicing, which overshadowed its canonical counterpart. After confirming a significant reduction in RNA levels caused by the G781A alteration in our ectopic assay, we probed the function of the G-rich sequence preceding the canonical exon 3 3’ splice site. Substituting the G-rich sequence with a typical pyrimidine-rich 3’ splice site sequence on C*03:23N resulted in a marked elevation in RNA levels, likely due to the enhanced preference for the canonical exon 3 3’ splice site over the alternate site. However, the same substitution led to a reduction in RNA levels for C*03:03:01. These findings suggested the dual roles of the G-rich sequence in RNA expression, and furthermore, underscore the importance of studying polymorphism effects within the framework of the entire gene, extending beyond conventional mini-gene reporter assays.

## 1 Introduction

The human major histocompatibility complex (MHC) region on chromosome 6, which contains a number of *HLA* (human leukocyte antigen) genes, is one of the most polymorphic genomic regions and also contains many other genes involved in immune response or sensory perception (1, 2). Classical HLA genes are essential for self versus non-self discrimination by presenting peptides with different sequences in their highly polymorphic peptide-binding pockets. The HLA region has the most disease associations compared to other region of the human genome (3–5), and over the years, extensive studies have been conducted to explore the biological consequences of HLA gene polymorphisms (2). In particular, these studies have focused on how these polymorphisms alter peptide binding specificity and the potential impact of such changes on immune recognition and disease susceptibility (6–8).

While many DNA polymorphisms of HLA genes result in amino acid changes in HLA proteins, some polymorphisms outside or within the coding exons do not alter the amino acids. Associations between genomic polymorphisms and HLA gene expression have been investigated using immunological measurements, real-time PCR, and more recently, high-throughput next-generation sequencing (NGS) (5, 9–20). Some of these investigations encompassed disease associations, such as the correlation of class I gene expression with ankylosing spondylitis (21), HIV control (22–25), Crohn’s disease (25), and acute graft-versus-host disease (GVHD) (26). Associations were also noted between class II gene expression and cystic fibrosis (27), hepatitis B virus infection (28), interstitial lung disease (29), and GVHD (30).

To elucidate the mechanisms driving the polymorphism-dependent differential expression of HLA genes, researchers have probed into the causal effects of DNA polymorphisms on gene expression utilizing mini-gene reporters that contain parts of the HLA genes. For example, Kulkarni et al. have proposed that alterations within the 3’ untranslated region (UTR) of *HLA-C* affects HIV viral load control via differences in binding affinity to the microRNA hsa-miR-148, a microRNA known to suppress HLA-C expression (23). Another investigation used a luciferase reporter assay to assess the effect of Oct-1 binding site polymorphisms and uncovered findings consistent with a causal relationship between these polymorphisms and HLA expression (31). However, the causal effect of polymorphisms on HLA gene expression remains largely unexplored. Even in cases where the reporter gene assay has been performed, the effect of a polymorphism(s) shown in the reporter assay does not necessarily predict the effect in the entire gene context, as the effect may vary depending on the polymorphic environment on individual HLA alleles.

In addition to binding of transcription factors and microRNA described above, RNA splicing is another process that can modify RNA expression. The classical MHC class I gene is organized into eight exons with distinct functional domains from exon 1 encoding the signal peptide, exons 2, 3 and 4 encoding the α1, α2 and α3 domains respectively, exon 5 encoding the transmembrane domain to the remaining three exons encoding the cytoplasmic tail. Depending on the loss or preservation of exons by alternative splicing, the structure and amount of RNAs would be altered, and the HLA protein could be membrane-bound, soluble, or completely degraded and inactivated. The purpose and mechanisms of alternative splicing of HLA class I genes for peptide presentation and regulation of T cell responses are still poorly understood. However, many null or low expression alleles of HLA class I genes have been reported including the *HLA-C* null allele, C*03:23N (32), which is the subject of the current work, and others (33–35). Although polymorphisms that influence RNA structure and abundance by modulating RNA splicing are anticipated to be widespread, they remain under-studied, with the exception of those leading to null or low-expression alleles.

In this study, we developed a novel ectopic expression assay to investigate the impact of HLA polymorphisms on RNA expression across the entire gene context for the class I *HLA-C* gene focusing mainly on three different alleles. First, we validated the assay by examining differential RNA expression levels for two HLA alleles, HLA-C*03:03:01 and C*04:01:01, which showed significantly different levels of RNA expression in RNA-seq association studies conducted by our team and others (10, 14, 15, 20). Subsequently, we employed the assay to examine the influence of a polymorphism that was implicated to a null phenotype of the HLA-C*03:23N at single nucleotide resolution, considering different polymorphic contexts of the gene.

## 2 Materials and methods

### 2.1 Plasmid constructs

To conduct the ectopic expression assay for different *HLA-C* gene alleles, we prepared HLA expression plasmids as follows. We used representative genomic DNA samples of two different *HLA-C* alleles, HLA-C*03:03:01 and C*04:01:01, which are reported to have significant differences in RNA levels as measured by our team and others (10, 14, 15, 20), and C*03:23N, which is a null allele identified by Shimizu et al. (32). These three *HLA-C* expression plasmids were constructed by inserting the entire *HLA-C* genomic segment of the known allele into a low copy-number expression vector pBkf polyA containing a replication origin derived from pET11c and a synthetic poly(A) site placed upstream of the insertion site. The three different *HLA-C* allelic segments were prepared by long-range PCR of genomic DNA samples (36). The PCR reactions were performed with PrimeSTAR GXL DNA Polymerase (Takara Bio) using the following parameters: denaturation at 94°C for 2 min, 30 cycles of denaturation at 98°C for 10 sec, and annealing and extension at 70°C for 3 min. To amplify the three *HLA-C* alleles, HLA-C_longF2 (5’-ACACGACCTGAGTCACATTAGCAGGA-3’) and HLA-C_longR3 (5’-GACAACAAAGGTCAGTTGAATGATCAGTG-3’) primers were used. The *HLA-C* PCR products were cloned into the pBkf polyA using In-Fusion HD Cloning Kit (Takara Bio). The inserted sequences in the plasmids were verified by Sanger sequencing. The CMV-green fluorescent protein (GFP) expression plasmid, used as a control, was derived from a CMV vector pCG and contained a sequence coding for enhanced green fluorescent protein (GFP). Single-nucleotide mutations and 10-bp replacement in the aforementioned *HLA*-assay plasmids were prepared by oligonucleotide mutagenesis using PrimeSTAR Mutagenesis Basal Kit (Takara Bio). To avoid unexpected mutations that might have been generated by mutagenesis procedures, the sequences encompassing the mutated regions were entirely confirmed by Sanger sequencing, and the sequenced segments were reinserted back into the original *HLA*-assay vectors. The detailed structures of the plasmids used in the study are available upon request.

### 2.2 Cell culture and transfection

K562 erythroleukemia cells were maintained in RPMI/1640 (Invitrogen) supplemented with 10% fetal bovine serum under 5% CO_2_ at 37°C. A total number of 1×10^7^ K562 cells were transfected with the HLA-C expression plasmid (4.5 µg) together with the GFP-control plasmid (0.5 µg) using a Neon transfection system (Invitrogen) in a 100 µl tip under the following conditions: voltage, 1,450 V; pulse, 3; width, 10 ms. The transfected cells were incubated for 48 hours before harvesting.

### 2.3 Quantitative reverse transcription PCR

In the quantitative reverse transcription PCR (rtPCR) assay, we designed primers for cDNA synthesis and amplification within a region identical among the three HLA-C alleles. This strategy ensures that variations in sequence among these alleles do not influence the efficiency of the reverse transcription and amplification reactions. Total RNA was isolated from transfected cell cultures using a Qiagen RNA extraction kit. To minimize the possible effects of sequence differences among the three *HLA-C* alleles on the efficiency of the reverse transcription and PCR, the primers were designed so that the hybridized target sequence and the amplicon had no sequence variation among the three alleles. Reverse transcription was performed with 100 nM qPCR_HLA-C-R1 primer (5’-GGCTTTACAAGCGATGAGAGACTCATCAGA-3’) and 1 µg of RNA using either SuperScript III First-Strand Synthesis system for RT-PCR kit (Invitrogen) in a volume of 20 µl or with ReverTra Ace qPCR RT kit (Toyobo) in a volume of 10 µl. The qPCR_HLA-C-R1 primer (5’-GGCTTTACAAGCGATGAGAGACTCATCAGA-3’) used for cDNA synthesis was designed to hybridize with the *HLA-C* target. We fortuitously found, however, that the qPCR_HLA-C-R1 primer not only initiates HLA-C cDNA synthesis but also GFP cDNA synthesis due to cross hybridization. Therefore, the qPCR_HLA-C-R1 primer was used for cDNA synthesis of both the *HLA-C* and *GFP* gene. The synthesized cDNAs were first diluted fivefold (1/5) and then were serially diluted in twofold steps up to 1/40, and 2 µl of each dilution was amplified using KOD SYBR qPCR Mix (Toyobo) in 10 µl reaction mixtures, containing forward and reverse primer at 200 nM. PCR was performed using the following parameters: denaturation at 98°C for 2 min, 40 cycles of denaturation at 98°C for 30 sec, and annealing and extension at 68°C for 30 sec. The primers used for PCR were as follows: qPCR_HLA-C-R1 (5’-GGCTTTACAAGCGATGAGAGACTCATCAGA-3’) and qPCR_HLA-C-F2 (5’-ATGTGTAGGAGGAAGAGCTCAGGTGGAAAA-3’), qPCR_HLA-C-R2 (5’-AGACTCATCAGAGCCCTGGGCACTGT-3’) and qPCR_HLA-C-F2 (5’-ATGTGTAGGAGGAAGAGCTCAGGTGGAAAA-3’), qPCR_GFP-F1 (5’-CCGACAAGCAGAAGAACGGCATCAAG-3’) and qPCR_GFP-R1 (5’-ACCATGTGATCGCGCTTCTCGTTG-3’).

**Figure 1:**
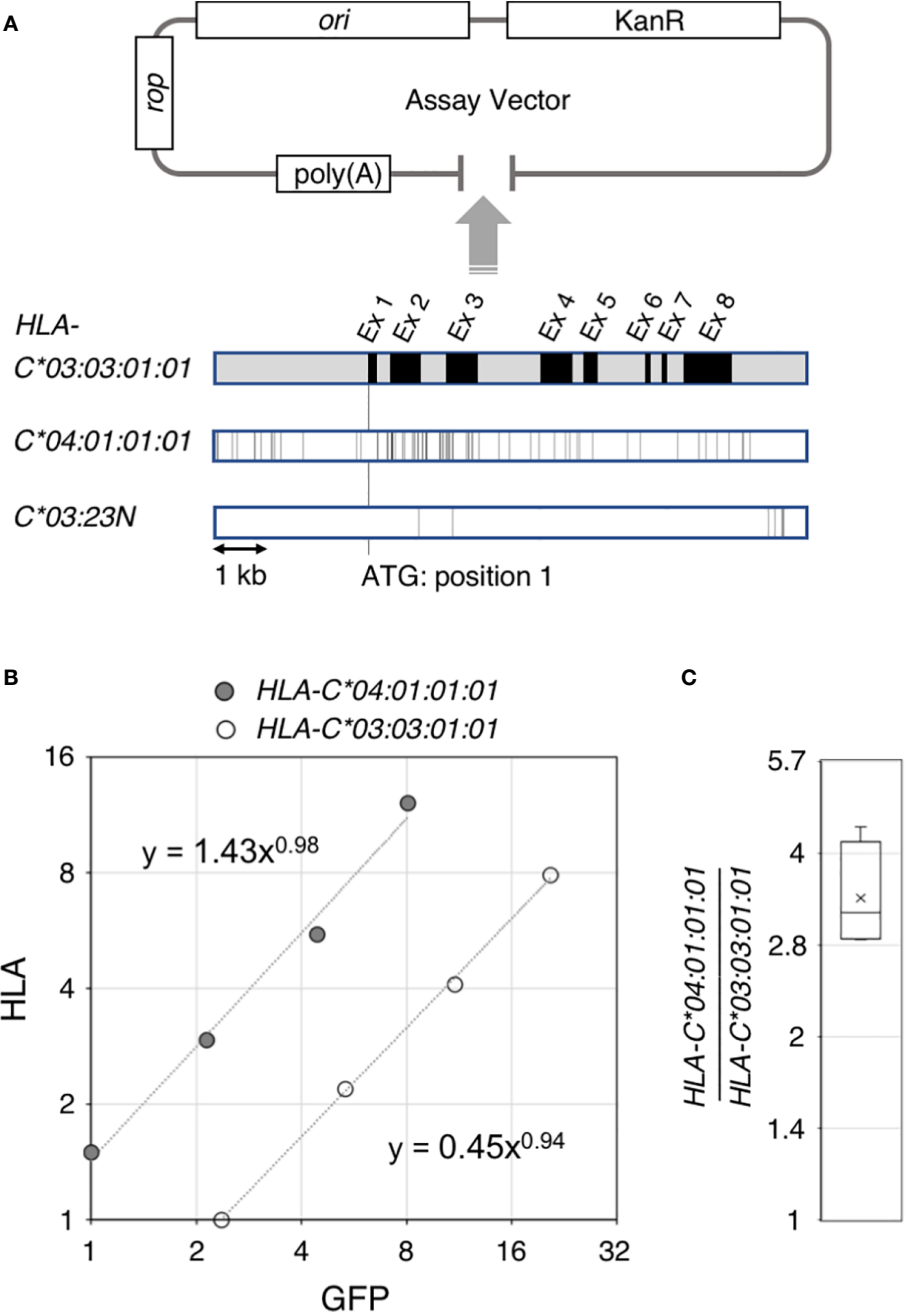
Ectopic expression assay of the *HLA-C* gene. Structure of the ectopic expression assay vector and comparison of the 4.6 kb gene segments of HLA-C*03:03:01:01, C*04:01:01:01 and C*03:23N are shown in **(A)**. A typical result of the ectopic expression assay is shown in **(B)**. HLA RNA levels for HLA-C* 04:01:01:01 and C*03:03:01:01estimated from quantitative reverse transcription PCR were plotted against internal control GFP RNA levels. Compilation of the HLA-C* 04:01:01:01 versus C*03:03:01:01 comparison from four independent experiments are shown in **(C)**. The bar and cross shown in the box represent average and median, respectively.

The results were quantified by the standard curve method. We first calculated the PCR efficiency of all the applicable samples in a plate excluding negative controls by plotting the Cr values against dilutions factors. The efficiency values of a given primer pair for a given PCR plate were estimated by averaging the corresponding PCR efficiencies calculated above. The initial relative target concentrations of each data point were calculated from the average PCR efficiency described above and Cr values. Finally, the *HLA-C* initial relative target concentrations were plotted against the *GFP* initial relative target concentrations; an example of such a plot is depicted in **Figure 1B**. From the graphs, we first derived exponential fit curves and functions to each dilution-series of data. To estimate the *HLA*-RNA level relative to the *GFP* control for a given sample series, we calculated the *HLA/GFP* values corresponding to the maximum and minimum GFP value and then assigned these two respective values to the function and averaged them.

### 2.4 Calculation and normalization of sequence read numbers (RNA levels) and analyses of splicing junction sequences

**Figure 2:**
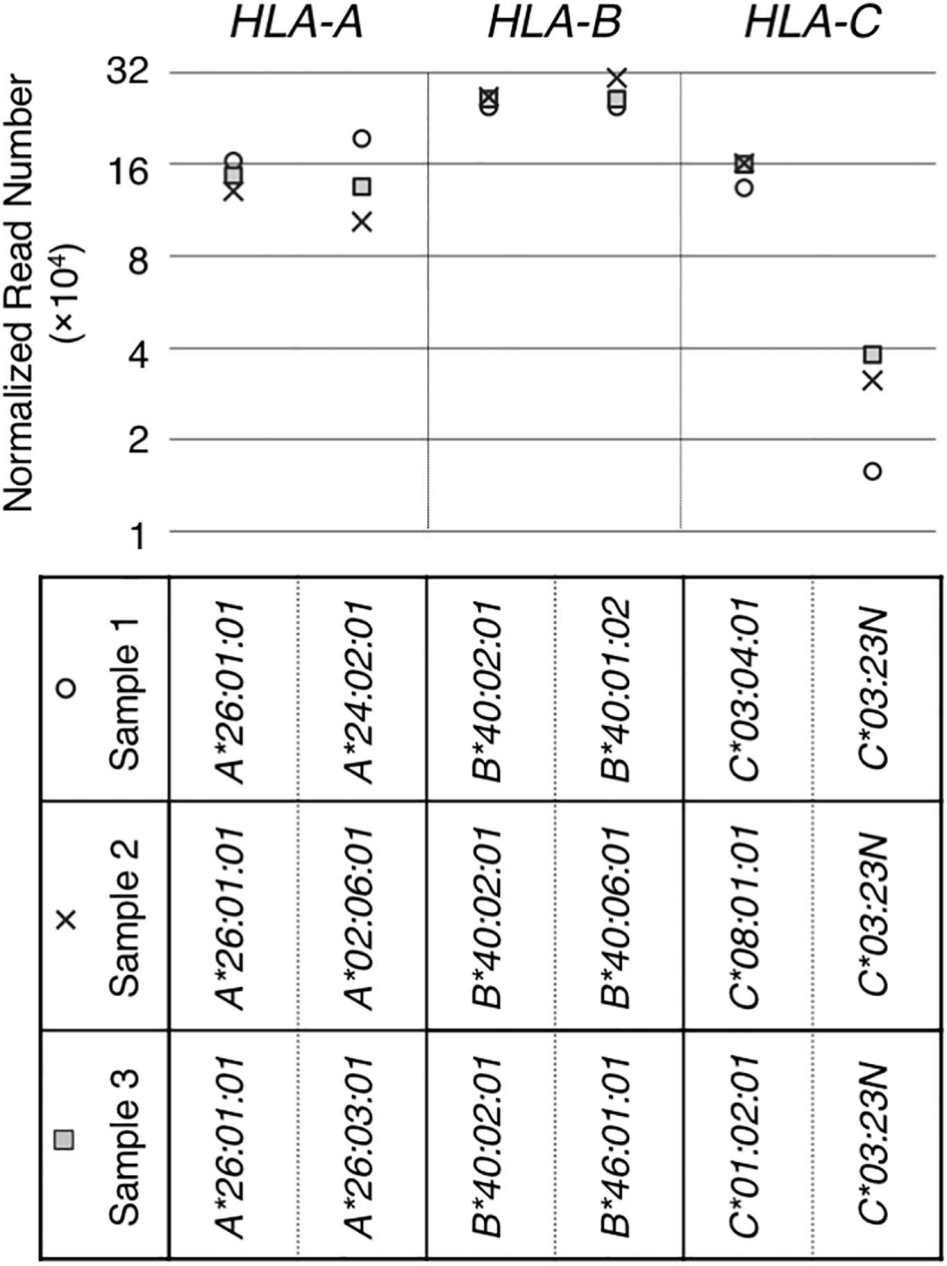
RNA levels quantitated from hybrid-capture RNA sequencing analyses. RNA levels of HLA class I genes/alleles in three samples (sample 1, 2 and 3), all containing the HLA-C*03:23N allele, were analyzed by the hybrid-capture RNA-seq assay (15). Normalized read numbers were plotted for individual *HLA-A*, *B* and *C* alleles in individual samples.

**Figure 3:**
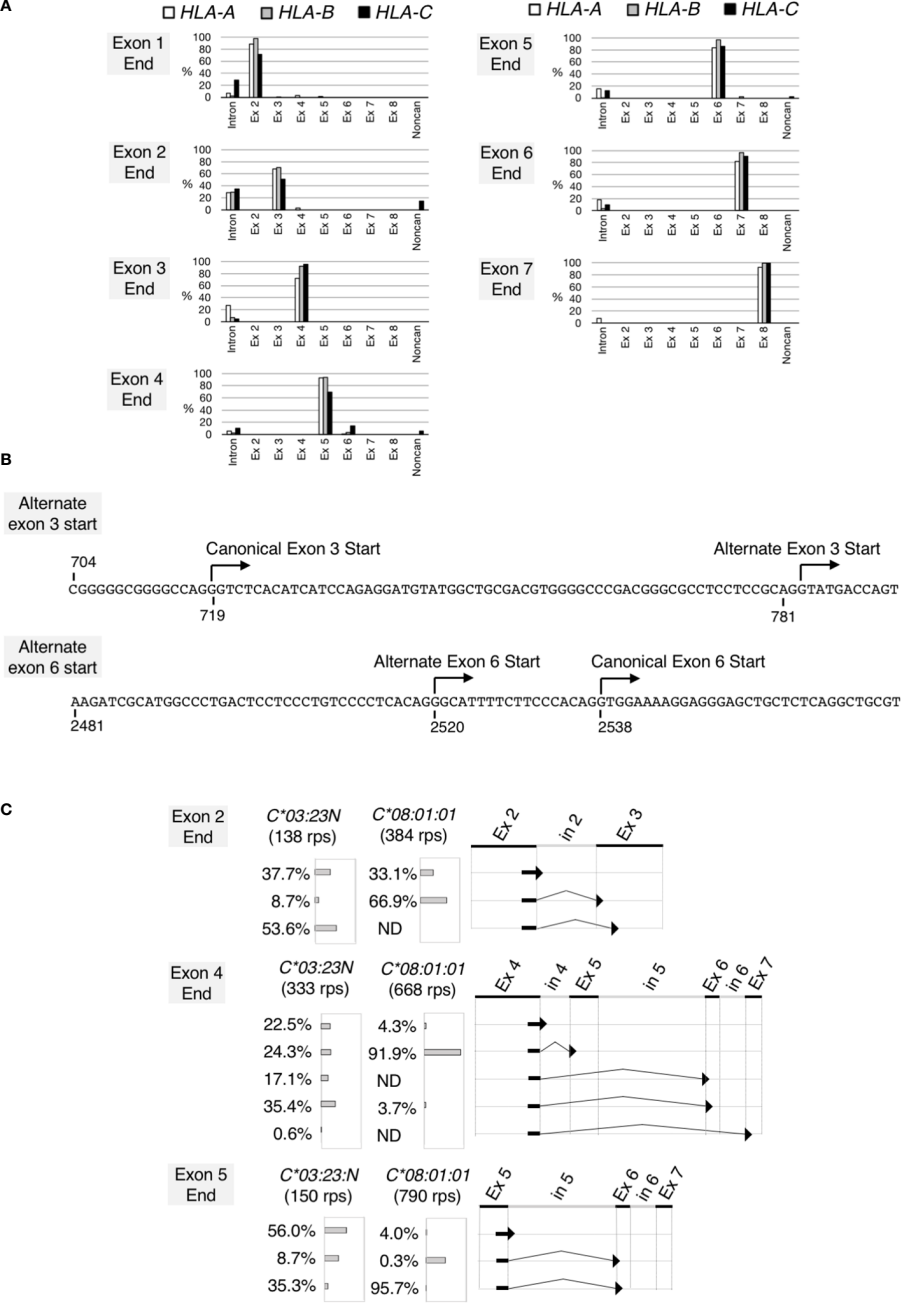
Noncanonical splicing revealed by analyses of RNA splicing profiles of the HLA class I genes. Splicing junctions were systematically analyzed using read pairs obtained from hybrid-capture RNA-seq analyses of the samples 2 described in **Figure 2**. In **(A)**, read pairs derived from the *HLA-A* (open bars), *B* (grey bars) and *C* (filled bars) genes were analyzed separately. Read pairs containing a given exon end were classified into read-pair groups according to the junction sequences that immediately follow the exon end. The proportions of the read-pair numbers belonging to each read-pairs group relative to total classifiable read-pair numbers at each exon end were plotted. Read-pairs groups that did not reach 0.5% were excluded from the plot. In **(B)** (top) the DNA sequence encompassing the canonical and alternate exon-3 start, (bottom) the DNA sequence encompassing the canonical and alternate exon-6 start. Both sequences are from HLA-C*03:23N. In **(C)**, splicing junctions at the end of exon 2, exon 4 and exon 5 were examined for HLA-C*03:23N and C*08:01:01 separately. Proportions (%) of junction sequences at each exon end were plotted.

Hybrid-capture RNA seq analyses were performed exactly as described by Yamamoto et al. (15), including derivation of normalized read numbers that is described in **Figure 2**, using the sample-set that we originally collected for our previous HLA-expression analyses (15). To analyze the splicing-junction sequences of sample 2 (see Results and **Figure 2** for sample 2 represented by A*26:01:01/A*02:06:01/B*40:02:01/B*40:06:01/C*08:01:01/C*03:23N), read pairs containing the *HLA* class I sequences were extracted from hybrid-capture RNA seq data by the methods described by Yamamoto el al (15). The *HLA-A*, *-B* and *-C* read-pair sequences were analyzed individually for splicing junctions, with the read pairs containing the 60-bp sequences corresponding to the 3’-ends of canonical exon 1 through exon 5, the 33-bp sequence containing the entire exon 6, or the 48-bp sequence containing the entire exon 7 sequence. The *HLA-A*, *-B* and *-C* RNA sequences were tagged and then the following tagged sequences were extracted: the 20-bp sequences that immediately follow the 60-bp tag sequences (for exon 1 to exon 5), the 47-bp sequences that immediately follow the 33-bp exon-6 tag sequence or the 32-bp that immediately follow the 48-bp exon-7 tag sequences. The extracted sequences were then aligned to DNA/RNA sequences corresponding to each *HLA* class I allele in the IPD-IMGT-HLA database (https://www.ebi.ac.uk/ipd/imgt/hla/), and the junction sequences at each 3’-end of the exons were classified (see **Figure 3A**). To analyze individually the exon 2 3’ends of the two *HLA-C* alleles, C*08:01:01 and C*03:23N, in sample 2 that have the identical 60-bp sequences, additional classification sequences corresponding to 10-bp sequences within the canonical exon 2 (i.e., position 128 through to 137 within the exon 3) were used.

## 3 Results

### 3.1 Ectopic expression assay of the *HLA-C* gene

Our ectopic expression assay to quantitate RNA levels of different *HLA-C* alleles in K562 erythroleukemia cells used a low copy-number plasmid vector as shown in **Figure 1A**. The 5.4-kb *HLA-C* segment was inserted into the pBkf polyA expression vector containing an upstream poly(A) site for eliminating transcripts that were read through the upstream vector region. In the current work, we compared three different *HLA-C* gene alleles–*03:03:01:01, 04:01:01:01* and *03:23N* (**Figure 1**). The *HLA*-assay plasmid was transfected into K562 cells, which showed no or very low levels of endogenous expression of the *HLA* class I genes together with a GFP internal control plasmid. In the quantitative rtPCR (reverse transcription PCR) assay, primers for cDNA synthesis and amplification were designed within a region that is identical among the three *HLA-C* alleles so that reverse transcription and amplification reaction are not affected by sequence differences among the three alleles (see MATERIALS AND METHODS).

### 3.2 RNA expression of HLA-C*03:03:01 and C*04:01:01

Our previous hybrid-capture RNA-seq association study (15) revealed an average 2-fold difference in the RNA levels between HLA-C*03:03:01 and C*04:01:01. To test if the difference in RNA-expression levels between these two alleles could be recapitulated in our ectopic expression assay, a 5.4-kb genomic segment containing HLA-C*03:03:01:01 or C*04:01:01:01 was cloned separately into the assay vector, shown in **Figure 1** (see panel A). RNAs from K562 cells transfected with either of the two *HLA*-assay plasmids, together with the GFP-control plasmid, were analyzed by the quantitative rtPCR assay. **Figure 1** shows a typical result of the assay (see panel B), in which *HLA* RNA levels were plotted against GFP levels from a 4-point serial dilution series of cDNA-input amounts in the rtPCR assay. In this case, the *HLA* expression of C*04:01:01:01 was 3.4-fold higher than HLA-C*03:03:01:01. The results were consistent among four independent assays, in which the fold difference between C*04:01:01:01 and C*03:03:01:01 was 3.4 in average, ranging between 2.9 and 4.3 (**Figure 1C**), demonstrating that the sequence differences between the two alleles caused the difference in the RNA levels in the assay. The background levels of HLA-RNA without an *HLA*-assay plasmid were three-magnitudes below the ectopic expression levels of C*03:03:01:01 showing that the assay can be used to analyze the effects of DNA-sequence differences on *HLA*-RNA expression levels with high sensitivities, despite the possible low levels of *HLA-C* RNA expression reported by Johnson (37).

### 3.3 RNA expression of the HLA-C*03:23N allele

The HLA-C*03:23N allele was described by Shimizu et al. as a null allele, originally based on low levels of cell-surface expression of the product (32). We searched for and identified three samples with HLA-C*03:23*N* in the sample-set that we originally collected for our previous HLA-expression analyses (15). These three HLA-C*03:23*N* samples, designated as samples 1, 2 and 3 in **Figure 2**, were used in our hybrid-capture RNA-seq method to compare the HLA-C*03:23N allele RNA levels to those of the other *HLA-C* alleles and to the *HLA-A* and *-B* alleles. The results displayed in **Figure 2** showed that RNA expression levels of this “null” allele ranged between 9.8% and 29% relative to the other *HLA-C* alleles included in the analysis (**Figure 2**).

We next examined RNA structures expressed by HLA-C*03:23N. We chose sample 2 in **Figure 2** for this analysis. All read pairs (RPs) containing the *HLA* class I sequences (760,229 RPs in total) were extracted, and analyzed systematically for splicing junction sequences. We first analyzed the *HLA-A*, *HLA-B* and *HLA-C* RPs individually. The *HLA* class-I RPs were classified as *HLA-A*, *HLA-B* or *HLA-*C based on the 60-bp tag sequences, typically, at each exon end (see Materials and methods for details). The compilation of the results, displaying the junction sequences following the 3’ends of individual exons, are presented in **Figure 3A**. In most cases, the sequences at the ends of exons are followed by the start of canonical exon sequences or by intron sequences that immediately follow the analyzed exon ends. We found, however, two apparent exceptions, both involving only *HLA-C*. In 14% of cases, the *HLA-C* exon 2 was spliced to a noncanonical exon-3 start at position 783 (see **Figure 3B**), consistent with the alternate 3’-splice site on HLA-C*03:23N described by Shimizu et al. (32). This noncanonical start at position 783 appears to be generated by the A variation at position 781 inside the canonical exon 3, corresponding to -2 (minus 2) position of the alternate 3’ site. The other exception was revealed at the end of exon 4. In 14% of cases, exon 4 was spliced to exon 6 instead of exon 5. In 5% of cases, exon 4 was spliced to a noncanonical exon start, which is located within intron 5 at position 2520, 18 nucleotide upstream of the exon-6 start (see **Figure 3B**). We also noted that among 21 exon-end analyses in total, there were 5 cases where unspliced RNAs were detected at over 20% of RPs, including all three exon 2-end cases. It is possible that higher ratios of unspliced RNAs are a reflection of the relatively less efficient and/or slower splicing mechanism at the 3’ end of exon 2 for the *HLA-A, B and C* genes.

To further analyze the noncanonical splicing detected for *HLA-C*, we examined RPs originated from the C*03:23N and C*08:01:01 alleles of the sample 2 separately. The results, shown in **Figure 3C**, indicated that the alternate exon-3 start from the exon 2 end was detected only on C*03:23N at 53.6%, but not on C*08:01:01. Similarly, exon-5 skipping from the exon 4 end was far more prominent on C*03:23N. Furthermore, the alternate exon-6 start at position 2520 from the exon 4 end was detected only on C*03:23N. We observed that aberrant or inefficient splicing to exon 6 also was significant when analyzed for junctions containing the exon-5 end. Of C*03:23N RNAs, only 35% of exon-5 was spliced to the canonical exon 6, 8.7% was spliced to the alternate exon 6, and 56% was unspliced. In contrast, 96% of exon-5 containing RNAs of C*08:01:01 was spliced to the canonical exon 6. These results showed that non-canonical splicing is particularly prominent on the HLA:C*03:23N allele, at the exon-2, exon-4 and exon-5 ends. The alternate exon-3 start is expected to result in a frameshift and formation of a premature termination codon, possibly leading to a truncated protein and also to instability of the transcripts (38). On the other hand, exon 5 is considered to contain a transmembrane domain (35), and the absence of a translated exon 5 might lead to the production of a soluble, but functional HLA protein.

### 3.4 Significance of nucleoside sequence variation at position 781 for *HLA-C* RNA expression

**Figure 4:**
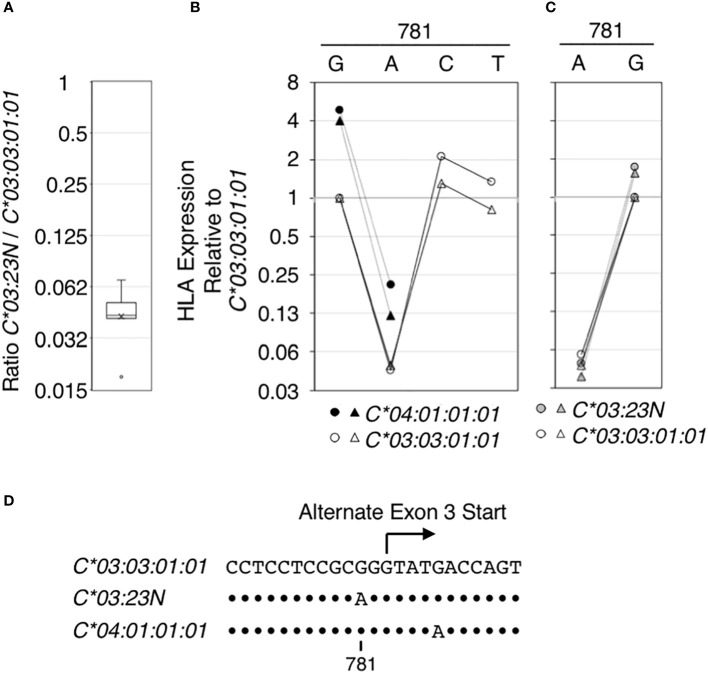
Regulatory effect of the nucleotide at position 781 on HLA RNA expression. The effect of the nucleotide sequence variation at position 781 in the context of HLA-C*03:03:01:01, C*03:23N and C* 04:01:01:01 on HLA RNA levels was examined using the ectopic expression assay. In **(A)**, RNA levels of HLA-C*03:23N relative to those of HLA-C*03:03:01:01 were measured in seven independent experiments, and the results were compiled and shown as a box-plot. Average and median (a bar and a cross in the box, respectively) are shown with an outlier (a small circle). In **(B)**, HLA RNA expression relative to that of HLA-C*03:03:01:01 was measured in two independent experiments for C*03:03:01:01 (open symbols) and C* 04:01:01:01 (filled symbols) that were mutated at position 781 as indicated. In **(C)**, HLA expression relative to that of HLA-C*03:03:01:01 (open symbols) was measured in two independent experiments for C*03:23N (filled gray symbols) that was mutated at position 781. In **(D)**, nucleotide sequences encompassing position 781 are shown for the three *HLA-C* alleles that were analyzed. Dots indicate positions where the sequences match those in C*03:03:01:01.

RNA levels of HLA:C*03:23N were examined in the ectopic-expression assay in parallel to HLA-C*03:03:01:01. The results, displayed in **Figure 4A**, showed that RNA levels of C*03:23N are an average 1/27th of those of C*03:03:01, indicating that the sequence differences between these two alleles (i.e., seven nucleotide positions in total) had led to a vast difference in RNA expression levels in the assay.

Among the seven sequence differences present between C*03:23N and C*03:03:01:01, the significance of G/A variation at position 781, possibly responsible for formation of the alternate 3’-splice site in C*03:23N, was tested directly by introducing the G to A mutation to C*03:03:01:01 and C*04:01:01:01. Data displayed in **Figure 4** showed that the G781A mutation in both alleles gave rise to an over 20-fold reduction in *HLA* RNA levels (see panel B). On the other hand, introducing the G781C or G781T mutation in *HLA*-C*03:03:01:01 did not significantly reduce the levels of *HLA* RNA. The results are consistent with the hypothesis that the G to A mutation at position 781, which is proceeded by pyrimidine-rich sequence, created an efficient 3’-splice site by replacing G with A residue at -2 position of the alternate 3’-splice site, and this led to skipping of the canonical exon-3 start and reduction of RNA expression.

We next performed a reciprocal test; the A residue at position 781 in HLA:C*03:23N was mutated to G. As shown in **Figure 4C**, the A to G mutation led to a greater than 20-fold increase in RNA levels of C*03:23N. Therefore, in the context of HLA-C*03:03:01:01, C*04:01:01:01 and C*03:23N, the nucleotide at 781 has a determining effect on RNA levels presumably by forming an efficient alternate 3’-splice site only if the position 781 is A, but not if it is G, C or T.

### 3.5 Differential effects of A781 variations on *HLA-C* RNA expression

**Figure 5:**
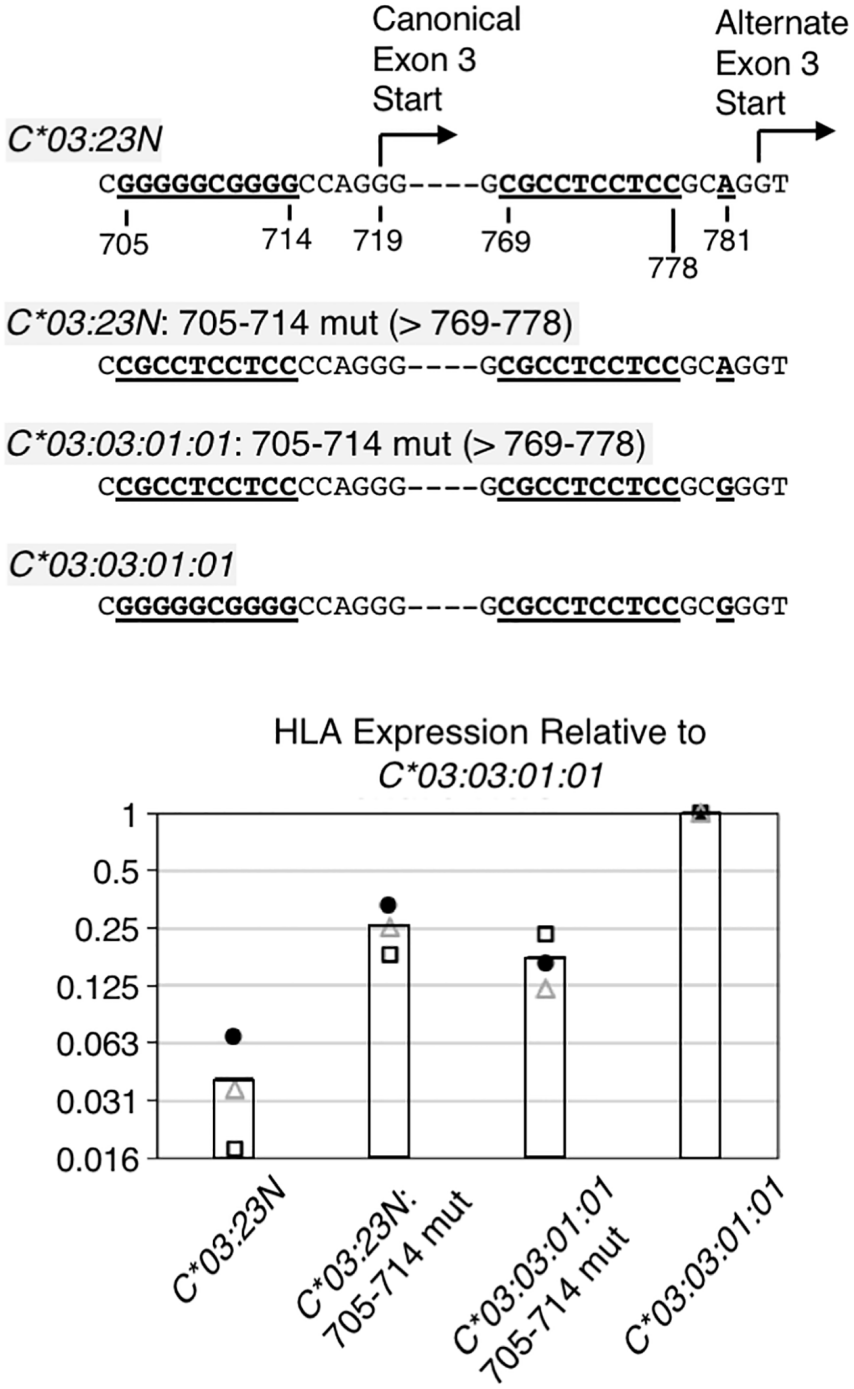
RNA analyses on the importance of the sequences preceding canonical exon-3 start. The 10-base pair G-rich sequence preceding the canonical exon-3 start was replaced with the 10-base pyrimidine-rich sequence in HLA-C*03:23N and C*03:03:01:01, and these replacement mutants were assayed for RNA levels by the ectopic expression assay. Structures of the replacement mutants are shown at the top. Note that the 704-to-783 sequence is different only at position 718 between the HLA-C*03:23N and C*03:03:01:01 allele. Results of three independent ectopic expression assays are shown at the bottom. RNA levels relative to the C*03:03:01:01 allele from three independent experiments are shown as filled circles, open triangles, and open rectangles. The bars represent average values of three independent assays.

In HLA-C*03:23N, RNAs containing the exon-2 end appear to be predominantly spliced to the alternate exon-3 start at position 783, but not to the canonical exon -3 start at position 719 (see **Figure 3C**). In RNA seq analyses, we found that the 3’-splice site at the exon-3 start appears to be relatively less efficient across the *HLA-A*, *B*, and *C* genes (see **Figure 3A**). In addition, it was noted that the 3’-splice site at the canonical exon-3 start does not contain a prototypical pyrimidine (C/T)-rich motif, but has a characteristic G-rich sequence. Based on these observations, we hypothesized that usage of the downstream alternate 3’-splice site, which is expected to lead to formation of a premature stop codon and reduction in RNA levels, is preferred in HLA-C*03:23N over the upstream canonical 3’-splice site at the exon-3 start because the latter is less efficient than the former 3’-splice site. We tested this possibility by replacing the 10-bp G-rich sequence (position 705-714) preceding the canonical exon-3 start with a 10-bp pyrimidine-rich sequence (position 769-778) preceding the alternate exon-3 start (**Figure 5**) so that the upstream canonical and downstream alternate 3’-splice site have an identical pyrimidine-rich sequence.

The results of ectopic assays of the C*03:23N replacement mutant (i.e., C*03:23N: 705-714 mut) in **Figure 5** show that, relative to parental C*03:23N, the replacement of G-rich 705-714 with pyrimidine-rich 769-778 led to nearly a 10-fold increase in RNA levels (**Figure 5**, see C*03:23*N*: 705-714 mut in panel B). This marked increase in RNA levels is consistent with the possibility that the 10-bp motif replacement has provided more efficient usage of the upstream canonical exon-3 start than the downstream alternate exon-3 start, resulting in an increased production of stable canonical mRNAs. We noted, however, that the RNA levels are still significantly lower than those of C*03:03:01:01.

For comparison, we next replaced the G-rich 705-714 sequence with the pyrimidine-rich 769-778 sequence in C*03:03:01:01, anticipating that introduction of a presumably more efficient 3’-splice site at the canonical exon-3 start may increase RNA expression. However, the results, described in **Figure 5** (see C*03:03:01:01: 705-714 mut) showed that this replacement resulted in a 4-fold decrease in RNA levels. This unexpected finding suggests that while the 705-714 G-rich sequence exerts a negative impact on *C03:23N* RNA expression, likely due to its inefficiency as a 3’-splice site, it plays a positive role in the RNA expression of *C03:03:01:01*. Furthermore, the comparison between *C03:23N*:705-714 mut and *C03:03:01:01*:705-714 mut revealed that, despite featuring A and G at position 781, respectively, they exhibited similar RNA expression levels. This suggests that the nucleotide at position 781 is not the sole determining factor of RNA levels between these two mutants. These findings, regarding the effect of the G-rich sequence and G781A, underscore how nucleotide polymorphisms or alterations can yield divergent effects based on the surrounding sequence context.

## 4 Discussion

In this study, we have presented a novel ectopic expression assay of the *HLA-C* gene that provides an experimental basis for investigating the causal effect of a polymorphism or combination of polymorphisms on RNA expression of the gene in the context of the entire gene. We first addressed the effect of the allelic sequence differences between HLA-C*03:03:01 and HLA-C*04:01:01 on RNA expression. We then applied the assay to a detailed analysis of a polymorphism found in a null allele HLA-C*03:23N, following a comprehensive analysis of the splicing patterns of C*03:23N.

In a number of studies using quantitative rtPCR and/or NGS, a few-fold difference in RNA expression levels between HLA-C*03:03:01 and C*04:01:01 was observed (10, 14, 15, 20). In our assay, we were able to show that in K562 cells, the DNA sequence differences between the two alleles are indeed causative in driving a few-fold difference in RNA expression. The DNA segments used for the ectopic assay of the two alleles differ at 78 positions. It remains to be investigated in the context of the entire gene how RNA expression of the two alleles is differentiated by DNA polymorphisms, including those suggested by association studies and reporter gene analyses (31).

For the C*03:23N null allele, we first analyzed the RNA expression and splicing patterns of C*03:23N by the capture RNA-seq method (15). The HLA-C*03:23N was identified as a null allele by Shimizu et al. (32). They suggested that the G781A polymorphism within exon 3 on C*03:23N generates an efficient 3’ splice site within exon 3. Using rtPCR-Sanger sequencing, they identified RNAs that skipped the canonical 3’ splice site at the start of exon 3 and were spliced into an alternate 3’ splice site within exon 3. This alternate exon 3 splicing is expected to introduce a frameshift and a premature stop codon, leading to a large reduction in cell surface expression of the C*03:23N protein (15).

Our capture RNA-seq analyses confirmed that the levels of C*03:23N RNA in three samples ranged between 9.8% and 29% relative to the levels of the other *HLA-C* alleles (see **Figure 2**). We next analyzed the splice junctions of C*03:23N and verified the occurrence of exon 3 alternative splicing. The amount of alternatively spliced products on C*03:23N was six times higher than those spliced at the canonical exon 3 start at position 719 (see **Figure 3**). In addition, we also noted instances of exon 5 skipping and alternative splicing involving a cryptic 3’ splice site within intron 5 at position 2520 on C*03:23N. These instances of alternative splicing were previously documented also in other *HLA-C* alleles or other HLA class I gene (33, 34, 39).

Since exon 5 is believed to encode the transmembrane region of HLA class I proteins, skipping past exon 5 would likely affect the cell surface expression of HLA class I proteins, leading to a reduction in protein presentation at the cell surface. However, in the current study, the relationship between alternate exon 3 start, exon 5 skipping and alternate exon 6 start in individual RNA molecules could not be resolved due to the short read lengths of our RNA sequence data. Nevertheless, alternate splicing at exon 3 and exon 5 skipping both occur frequently in C*03:23N, suggesting a plausible relationship between alternative splicing and exonic skipping. We also note that a premature termination codon has been reported to affect splicing patterns in some cases (38). Overall, comprehensive analyses of HLA gene splicing patterns, as described in this study, have been scarce, but are important for a better understanding of the effects of polymorphisms on RNA expression through transcription and/or splicing of the HLA genes.

Experimental verification of the causal effect of the G781A polymorphism on RNA expression in the HLA-C*03:23N null allele was conducted using the ectopic assay. In all polymorphic contexts of HLA-C*03:23N, HLA-C*03:03:01:01, and C*04:01:01:01, RNA levels of the A781 variants were consistently less than 1/20th of the G781 variants. Therefore, the substantial impact of A781 is not confined to the specific sequence context of the C*03:23N allele. Moreover, in line with the suggestion that A781 corresponds to the -2 position of an alternate 3’ splice site, the G781A, but not the G781T or G781C variants, showed a vastly reduced RNA levels. These results suggest that G781A leads to a substantial reduction in RNA levels, provided that the downstream 3’ splice site generated by the G781A polymorphism is preferentially utilized over the 3’ splice site. Indeed, analyses of all splice junctions in RNA-seq data for *HLA-A*, *B*, and *C* genes indicated that splicing between the end of exon-2 and the G-rich non-prototypical 3’ splice site at the start of exon 3 is the least efficient among all splice junctions in each of the *HLA-A*, *-B*, and *-C* genes (refer to **Figure 3**). In contrast, the alternative 3’ site produced by the G781A polymorphism features a pyrimidine-rich sequence, characteristic of a prototypical 3’ splice site, and is expected to function efficiently (32).

The impact of the two tandem 3’ splice sites in C*03:23N exon 3, including the upstream G-rich site at the canonical start of exon 3 and the downstream pyrimidine-rich site generated by the G781A polymorphism, on RNA expression levels was evaluated by mutating the G-rich site (position 715-714) to the pyrimidine-rich site (position 769-778). RNA expression levels of this 715-714 mutant were nearly 10-fold higher than those of the parental C*03:23N, supporting the notion that introducing a presumably more efficient 3’ splice site at the start of exon 3 results in improved use of the upstream 3’ splice site.

These analyses also revealed that the RNA levels of the 705-714 nucleotide replacement mutant in C*03:23N did not reach the levels observed in C*03:03:01:01. Furthermore, introducing the same replacement mutation into C*03:03:01:01 resulted in a 5-fold reduction in RNA levels. One possible explanation for this puzzling result could be a transcriptionally positive role of the G-rich 3’ splice site sequence. This transcriptional role may elucidate why the G-rich 3’ site has been evolutionarily conserved despite its inefficiency as a 3’ splice site. Although not explored in the *HLA-C* gene, intronic enhancers have been observed in numerous genes (40–42), and genome-wide analyses of transcription factor binding site (TFBS) clusters suggest that a significant proportion of these clusters are located in intronic regions (43). Several transcription factors are predicted to bind to the G-rich sequence, including SP1, whose binding consensus sequence (G/T)GGGCGG(G/A)(G/A)(C/T) perfectly matches the G-rich sequence.

Importantly, these results highlight that while the 715-714 replacement mutation exhibits an up-mutation effect in C*03:23N, it has a contrasting down-mutation impact on C*03:03:01:01. While these observed effects are not attributed to natural polymorphisms, they demonstrate that the influence of a specific polymorphism can theoretically vary to a significant extent and potentially lead to opposing effects depending on the allelic context. Moreover, we presented another example of differential effects of a polymorphism that is dependent on the sequence context; the nucleotide at position 781 is critical on C*03:03:01:01, C*03:23N and C*04:01:01:01, but the effect was minimal on the two 705-714 replacement mutants, C*03:23N: 705-714 mut and C*03:03:01:01: 705-714 mut. These findings underscore the significance of analyzing the effects of polymorphisms in the context of entire genes, as demonstrated in this study, beyond the realm of mini-gene reporter assays that use gene fragments. Such an approach is critical for elucidating how polymorphisms in the *HLA* genes influence their function via their effects on gene product abundance, structure, and regulation.

## Data availability statement

The capture RNA-seq data used in the study will be stored and maintained on a data server at Tokai University School of Medicine for at least 5 years and will be made available to interested parties upon request for validating the findings described in the paper. However, if anybody wants to use the raw NGS data beyond evaluating the current work, a written permission will need to be obtained from The Japanese Data Center for Hematopoietic Transplantation (JDCHCT: http://www.jdchct.or.jp/en/outline/) for such usage. Requests to access the datasets should be directed to TSh, tshiina@is.icc.u-tokai.ac.jp.

## Ethics statement

The studies involving humans were approved by Institutional Review Board for Clinical Research,Tokai University Ethics committee. The studies were conducted in accordance with the local legislation and institutional requirements. The human samples used in this study were acquired from primarily isolated as part of your previous study for which ethical approval was obtained. Written informed consent for participation was not required from the participants or the participants’ legal guardians/next of kin in accordance with the national legislation and institutional requirements.

## Author contributions

AM: Conceptualization, Data curation, Formal analysis, Funding acquisition, Investigation, Methodology, Validation, Visualization, Writing – original draft, Writing – review & editing. SS: Conceptualization, Investigation, Methodology, Resources, Software, Writing – review & editing. AS: Methodology, Writing – review & editing. TSa: Methodology, Writing – review & editing. MT: Conceptualization, Formal analysis, Investigation, Supervision, Visualization, Writing – original draft, Writing – review & editing. JK: Conceptualization, Formal analysis, Investigation, Writing – original draft, Writing – review & editing. TSh: Conceptualization, Data curation, Formal analysis, Funding acquisition, Investigation, Project administration, Supervision, Writing – original draft, Writing – review & editing.

## References

[1] Beck S Geraghty D Inoko H Rowen L Aguado B Bahram S. Complete sequence and gene map of a human major histocompatibility complex. Nature (1999) 401(6756):921–3. doi: 10.1038/44853 10553908

[2] Trowsdale J Knight JC. Major histocompatibility complex genomics and human disease. Annu Rev Genomics Hum Genet (2013) 14:301–23. doi: 10.1146/annurev-genom-091212-153455 PMC4426292 23875801

[3] Consortium WTCC. Genome-wide association study of 14,000 cases of seven common diseases and 3,000 shared controls. Nature (2007) 447(7145):661–78. doi: 10.1038/nature05911 PMC2719288 17554300

[4] Fellay J Shianna KV Ge D Colombo S Ledergerber B Weale M. A whole-genome association study of major determinants for host control of hiv-1. Science (2007) 317(5840):944–7. doi: 10.1126/science.1143767 PMC1991296 17641165

[5] Petersdorf EW O'HUigin C. The mhc in the era of next-generation sequencing: implications for bridging structure with function. Hum Immunol (2019) 80(1):67–78. doi: 10.1016/j.humimm.2018.10.002 30321633 PMC6542361

[6] Gough SC Simmonds MJ. The Hla region and autoimmune disease: associations and mechanisms of action. Curr Genomics (2007) 8(7):453–65. doi: 10.2174/138920207783591690 PMC2647156 19412418

[7] Blackwell JM Jamieson SE Burgner D. Hla and infectious diseases. Clin Microbiol Rev (2009) 22(2):370–85. doi: 10.1128/CMR.00048-08 PMC2668228 19366919

[8] Medhasi S Chantratita N. Human leukocyte antigen (Hla) system: genetics and association with bacterial and viral infections. J Immunol Res (2022) 2022:9710376. doi: 10.1155/2022/9710376 35664353 PMC9162874

[9] Bettens F Brunet L Tiercy JM. High-allelic variability in Hla-C mrna expression: association with Hla-extended haplotypes. Genes Immun (2014) 15(3):176–81. doi: 10.1038/gene.2014.1 24500399

[10] Bettens F Ongen H Rey G Buhler S Calderin Sollet Z Dermitzakis E. Regulation of Hla class I expression by non-coding gene variations. PloS Genet (2022) 18(6):e1010212. doi: 10.1371/journal.pgen.1010212 35666741 PMC9170083

[11] Ramsuran V Kulkarni S O'Huigin C Yuki Y Augusto DG Gao X. Epigenetic regulation of differential Hla-a allelic expression levels. Hum Mol Genet (2015) 24(15):4268–75. doi: 10.1093/hmg/ddv158 PMC4492392 25935001

[12] Rene C Lozano C Villalba M Eliaou JF. 5' and 3' Untranslated regions contribute to the differential expression of specific Hla-a alleles. Eur J Immunol (2015) 45(12):3454–63. doi: 10.1002/eji.201545927 26399450

[13] Pan N Lu S Wang W Miao F Sun H Wu S. Quantification of classical Hla class I mrna by allele-specific, real-time polymerase chain reaction for most han individuals. HLA (2018) 91(2):112–23. doi: 10.1111/tan.13186 29178661

[14] Aguiar VRC Cesar J Delaneau O Dermitzakis ET Meyer D. Expression estimation and eqtl mapping for Hla genes with a personalized pipeline. PloS Genet (2019) 15(4):e1008091. doi: 10.1371/journal.pgen.1008091 31009447 PMC6497317

[15] Yamamoto F Suzuki S Mizutani A Shigenari A Ito S Kametani Y. Capturing differential allele-level expression and genotypes of all classical Hla loci and haplotypes by a new capture rna-seq method. Front Immunol (2020) 11:941. doi: 10.3389/fimmu.2020.00941 32547543 PMC7272581

[16] Johansson T Yohannes DA Koskela S Partanen J Saavalainen P. Hla rna sequencing with unique molecular identifiers reveals high allele-specific variability in mrna expression. Front Immunol (2021) 12:629059. doi: 10.3389/fimmu.2021.629059 33717155 PMC7949471

[17] Panigrahi A O'Malley BW. Mechanisms of enhancer action: the known and the unknown. Genome Biol (2021) 22(1):108. doi: 10.1186/s13059-021-02322-1 33858480 PMC8051032

[18] Cornaby C Montgomery MC Liu C Weimer ET. Unique molecular identifier-based high-resolution Hla typing and transcript quantitation using long-read sequencing. Front Genet (2022) 13:901377. doi: 10.3389/fgene.2022.901377 35879986 PMC9308011

[19] Johansson T Partanen J Saavalainen P. Hla allele-specific expression: methods, disease associations, and relevance in hematopoietic stem cell transplantation. Front Immunol (2022) 13:1007425. doi: 10.3389/fimmu.2022.1007425 36248878 PMC9554311

[20] Aguiar VRC Castelli EC Single RM Bashirova A Ramsuran V Kulkarni S. Comparison between qpcr and rna-seq reveals challenges of quantifying Hla expression. Immunogenetics (2023) 75(3):249–62. doi: 10.1007/s00251-023-01296-7 PMC9883133 36707444

[21] Cauli A Dessole G Fiorillo MT Vacca A Mameli A Bitti P. Increased level of Hla-B27 expression in ankylosing spondylitis patients compared with healthy Hla-B27-positive subjects: A possible further susceptibility factor for the development of disease. Rheumatol (Oxford) (2002) 41(12):1375–9. doi: 10.1093/rheumatology/41.12.1375 12468816

[22] Thomas R Apps R Qi Y Gao X Male V O'HUigin C. Hla-C cell surface expression and control of hiv/aids correlate with a variant upstream of Hla-C. Nat Genet (2009) 41(12):1290–4. doi: 10.1038/ng.486 PMC2887091 19935663

[23] Kulkarni S Savan R Qi Y Gao X Yuki Y Bass SE. Differential microrna regulation of Hla-C expression and its association with hiv control. Nature (2011) 472(7344):495–8. doi: 10.1038/nature09914 PMC3084326 21499264

[24] Apps R Carrington M. Response to comment on "Influence of Hla-C expression level on hiv control". Science (2013) 341(6151):1175. doi: 10.1126/science.1241854 PMC6314016 24031003

[25] Kulkarni S Qi Y O'HUigin C Pereyra F Ramsuran V McLaren P. Genetic interplay between Hla-C and mir148a in hiv control and crohn disease. Proc Natl Acad Sci U.S.A. (2013) 110(51):20705–10. doi: 10.1073/pnas.1312237110 PMC3870724 24248364

[26] Petersdorf EW Gooley TA Malkki M Bacigalupo AP Cesbron A Du Toit E. Hla-C expression levels define permissible mismatches in hematopoietic cell transplantation. Blood (2014) 124(26):3996–4003. doi: 10.1182/blood-2014-09-599969 25323824 PMC4271183

[27] Hofer TP Frankenberger M Heimbeck I Burggraf D Wjst M Wright AK. Decreased expression of Hla-dq and Hla-dr on cells of the monocytic lineage in cystic fibrosis. J Mol Med (Berl) (2014) 92(12):1293–304. doi: 10.1007/s00109-014-1200-z 25146850

[28] Thomas R Thio CL Apps R Qi Y Gao X Marti D. A novel variant marking Hla-dp expression levels predicts recovery from hepatitis B virus infection. J Virol (2012) 86(12):6979–85. doi: 10.1128/JVI.00406-12 PMC3393572 22496224

[29] Odani T Yasuda S Ota Y Fujieda Y Kon Y Horita T. Up-regulated expression of Hla-drb5 transcripts and high frequency of the Hla-drb5*01:05 allele in scleroderma patients with interstitial lung disease. Rheumatol (Oxford) (2012) 51(10):1765–74. doi: 10.1093/rheumatology/kes149 22723597

[30] Petersdorf EW Malkki M O'HUigin C Carrington M Gooley T Haagenson MD. High Hla-dp expression and graft-versus-host disease. N Engl J Med (2015) 373(7):599–609. doi: 10.1056/NEJMoa1500140 26267621 PMC4560117

[31] Vince N Li H Ramsuran V Naranbhai V Duh FM Fairfax BP. Hla-C level is regulated by a polymorphic oct1 binding site in the Hla-C promoter region. Am J Hum Genet (2016) 99(6):1353–8. doi: 10.1016/j.ajhg.2016.09.023 PMC5142108 27817866

[32] Shimizu M Kuroda Y Uchida M Takada S Kamada H Takahashi D. A new Hla-C allele with an alternative splice site in exon 3: Hla-C*03:23n. HLA (2020) 95(6):555–60. doi: 10.1111/tan.13832 32034867

[33] Krangel MS. Secretion of Hla-a and -B antigens via an alternative rna splicing pathway. J Exp Med (1986) 163(5):1173–90. doi: 10.1084/jem.163.5.1173 PMC2188102 3701253

[34] Tijssen HJ Sistermans EA Joosten I. A unique second donor splice site in the intron 5 sequence of the Hla-a*11 alleles results in a class I transcript encoding a molecule with an elongated cytoplasmic domain. Tissue Antigens (2000) 55(5):422–8. doi: 10.1034/j.1399-0039.2000.550504.x 10885562

[35] Voorter CE Gerritsen KE Groeneweg M Wieten L Tilanus MG. The role of gene polymorphism in Hla class I splicing. Int J Immunogenet (2016) 43(2):65–78. doi: 10.1111/iji.12256 26920492

[36] Shiina T Suzuki S Ozaki Y Taira H Kikkawa E Shigenari A. Super high resolution for single molecule-sequence-based typing of classical Hla loci at the 8-digit level using next generation sequencers. Tissue Antigens (2012) 80(4):305–16. doi: 10.1111/j.1399-0039.2012.01941.x 22861646

[37] Johnson DR. Differential expression of human major histocompatibility class I loci: Hla-a, -B, and -C. Hum Immunol (2000) 61(4):389–96. doi: 10.1016/s0198-8859(99)00186-x 10715516

[38] Hwang J Kim YK. When a ribosome encounters a premature termination codon. BMB Rep (2013) 46(1):9–16. doi: 10.5483/bmbrep.2013.46.1.002 23351378 PMC4133823

[39] Ehlers FAI Olieslagers TI Groeneweg M Bos GMJ Tilanus MGJ Voorter CEM. Polymorphic differences within Hla-C alleles contribute to alternatively spliced transcripts lacking exon 5. HLA (2022) 100(3):232–43. doi: 10.1111/tan.14695 PMC9546215 35650170

[40] Bergman Y Rice D Grosschedl R Baltimore D. Two regulatory elements for immunoglobulin kappa light chain gene expression. Proc Natl Acad Sci U.S.A. (1984) 81(22):7041–5. doi: 10.1073/pnas.81.22.7041 PMC392072 6438631

[41] Muller F Chang B Albert S Fischer N Tora L Strahle U. Intronic enhancers control expression of zebrafish sonic hedgehog in floor plate and notochord. Development (1999) 126(10):2103–16. doi: 10.1242/dev.126.10.2103 10207136

[42] Panigrahi AK Foulds CE Lanz RB Hamilton RA Yi P Lonard DM. Src-3 coactivator governs dynamic estrogen-induced chromatin looping interactions during transcription. Mol Cell (2018) 70(4):679–94 e7. doi: 10.1016/j.molcel.2018.04.014 29775582 PMC5966282

[43] Chen H Li H Liu F Zheng X Wang S Bo X. An integrative analysis of tfbs-clustered regions reveals new transcriptional regulation models on the accessible chromatin landscape. Sci Rep (2015) 5:8465. doi: 10.1038/srep08465 25682954 PMC4329551

